# The complete mitogenome of *Helixpomatia* and the basal phylogeny of Helicinae (Gastropoda, Stylommatophora, Helicidae)

**DOI:** 10.3897/zookeys.827.33057

**Published:** 2019-03-05

**Authors:** Ondřej orábek, Adam Petrusek, Michail Rovatsos

**Affiliations:** 1 Department of Ecology, Faculty of Science, Charles University, Viničná 7, CZ-12844 Prague 2, Czechia Charles University Prague Czech Republic

**Keywords:** Allognathini, biogeography, Helicini, land snail, Mediterranean, mitochondrial genome, Otalini, phylogeny, Thebini

## Abstract

A complete mitochondrial genome of the Roman snail *Helixpomatia* Linnaeus, 1758 has been sequenced. The length and gene order correspond to that of other available helicid mitogenomes. We used the mitogenome sequence to reappraise the relationships among the four presumed principal groups of the helicid subfamily Helicinae. The results support the idea that the subfamily is divided between two western Palaearctic diversification centres: Iberian Peninsula and western Maghreb in the west, and Anatolia, the Aegean and Caucasus in the east. One group, the tribe Helicini, diversified in the east and the remaining three currently recognised tribes in the west. However, the exact relationships among lineages of the non-Helicini tribes could not be resolved.

## Introduction

Phylogenetic research on land snails has been thus far dominated by studies based on mitochondrial markers, mostly partial sequences of the *cox1* (COI) and *rrnL* (16S rRNA) genes, not exceeding 2000 bp in total. These genes dominate not only in terms of the amount of sequences generated, but usually also by the variability of the sequences used if combined with sequences of nuclear loci. Although modern techniques targeting many loci simultaneously begin to advance also into the phylogenetics of pulmonate land snails (Teasdale et al. 2016), the mitochondrial markers will likely continue to be used not only due to relatively low costs and accessibility of the methods, but especially thanks to the wealth of previously published data. Mitochondrial genome sequences may then become handy for primer design, to facilitate assembly of further mitochondrial genomes from outputs of high-throughput sequencing, or directly as the data for phylogenetic analyses. In fact, one of the best resolved backbone phylogenies of Stylommatophora to date was reconstructed from mitochondrial genome sequences (Groenenberg et al. 2017).

We sequenced a complete mitogenome of *Helixpomatia* Linnaeus, 1758, a large and common European edible land snail species, to provide a reference for further work focused on the family Helicidae Rafinesque, 1815. We then used other helicid mitogenomic sequences to evaluate the support for the basal branching order in the subfamily Helicinae, which was reported by Neiber and Hausdorf (2015) from analyses based on partial sequences of *rrnL* and *cox1* genes along with part of the nuclear rRNA gene cluster (5.8S rRNA, internal transcribed spacer 2, 28S rRNA).

The subfamily Helicinae includes several charismatic species, which are large, common, and edible. As the most prominent, we may mention *Cepaeanemoralis* (Linnaeus, 1758), famous for evolutionary studies of its colour polymorphism (e.g. Cain and Sheppard 1950, Jones et al. 1977, Silvertown et al. 2011), *Cornuaspersum* (Müller, 1774), the most commonly reared and consumed helicid species which became a pest in several regions around the globe (e.g. Rees 1955, Ansart et al. 2009), and *Helixpomatia*, a characteristic snail of Central Europe and the type species of the family’s type genus. Each of these three species represents a different lineage of the three presumed clades constituting the Helicinae, which were proposed and assigned a tribe status in the first comprehensive molecular phylogenetic study of Helicoidea by Razkin et al. (2015). *Cepaea* represents Allognathini Westerlund, 1902, *Helix*Helicini Rafinesque, 1815, and *Cornu*Otalini Pfeffer, 1930. Razkin et al. (2015) also recognized a fourth tribe, the monotypic Thebini Wenz, 1923 represented by *Theba* Risso, 1826. The phylogenetic position of *Theba* has been contentious (Perrot 1939, Gittenberger and Ripken 1987, Bouchet and Rocroi 2005, Schileyko 2013, Razkin et al. 2015), but *Helix*, *Cornu* and *Cepaea* were generally considered closer to each other than to *Theba*, for which a separate tribe or even subfamily has been erected.

Neiber and Hausdorf (2015) used a more informative dataset than Razkin et al. (2015) and found *Cepaea, Cornu* and *Theba* to be more closely related to each other than to *Helix* and some other eastern Mediterranean genera. Their results suggest a principal biogeographic division within Helicinae, running approximately along the Apennine Peninsula and separating two equally old clades with centre of diversification in the western (*Cornu, Cepaea* and *Theba*, with their respective relatives) and the eastern (*Helix* and relatives; Korábek et al. 2015) Mediterranean basin, respectively. Considering the Paleogene/Neogene palaeogeographic evolution of the western Mediterranean (Giusti and Manganelli 1984, Rosenbaum et al. 2002, Advokaat et al. 2014, van Hinsbergen et al. 2014), it may be expected that the western taxa are indeed more closely related to each other.

Alternatively, the eastern group may be an offshoot of the western diversity, if the western group is paraphyletic. The systematic concept and monophyly of Otalini remains an open issue, as they (in the sense of Neiber and Hausdorf 2015) may be paraphyletic to both Thebini and Allognathini, and the east-west divide has not been unambiguously supported. The absence of an east-west divide in the phylogeny would lend support to a Western European origin of the whole subfamily Helicinae and to the north-western Maghreb being a refugium of its oldest and perhaps phylogenetically most distinctive lineages. Apart from biogeographic implications, the branching order at the base of the Helicinae has for us also a practical significance for the selection of appropriate outgroup taxa for analyses of relationships between *Helix* and related genera.

There are already published complete mitochondrial genome sequences of *Ce.nemoralis* (Terrett et al. 1996; NCBI accession number NC_001816), *Co.apersum* (Gaitán-Espitia et al. 2013; NCBI accession number NC_021747) and *Thebapisana* (Müller, 1774) (Wang et al. 2018b; Genbank accession number MH362760), as well as of a convenient outgroup species *Cylindrusobtusus* (Draparnaud, 1805) (Groenenberg et al. 2012; NC_017872). Combined with the newly sequenced mitogenome of *Helixpomatia* they offered an opportunity to verify the relationships between the four Helicinae tribes.

## Methods

### Sequencing and annotation

As a starting point we utilised mitochondrial sequences obtained from transcriptome sequencing (mRNA-Seq) performed for a different project, which included also a single *H.pomatia* individual (from Rožmitál pod Třemšínem, Czechia). In detail, 20 mg of foot tissue was homogenised with MagNA Lyser (Roche) and total RNA was extracted using the standard Trizol reagent protocol (Thermo Fisher Scientific). The barcoded and stranded mRNA-sequencing libraries were prepared using the Illumina TruSeq mRNA v2 sample preparation kit (Illumina, San Diego, CA, USA). The libraries were loaded on an Illumina NextSeq 500 sequencer and 75 bp were sequenced uni-directionally, resulting in approx. 87 million reads. The raw Illumina reads were trimmed for adapters and low-quality bases in GENEIOUS R7.1 (Kearse et al. 2012) with the trim utility using default parameters. Reads with a length of less than 50 bp were removed from the dataset. The trimmed reads were checked for quality in FASTQC (Andrews 2010) and MULTIQC (Ewels et al. 2016), and mapped with GENEIOUS R7.1 to the mitogenome of *Co.aspersum*. The partial sequences of 12 protein coding genes (all but *atp8*) of *H.pomatia* (GenBank acc. nos. MK400678-MK400689) were extracted from the alignment and were used to design primers for PCR amplification of the mitogenome of *H.pomatia* in few long overlapping fragments.

For amplification we used another individual sharing an identical *cox1* sequence with the transcriptome data. The sample originated from Huldsessen, Bavaria, Germany (48.3978N, 12.7084E) and the shell voucher is deposited in the National Museum, Prague, lot P6M 29637. We designed specific primers with Primer BLAST (Ye et al. 2012) based on the transcriptome sequences and previous 16S data. Then, we amplified the mitogenome in several overlapping segments of ca. 4000–6000 bp with Platinum SuperFi proof-reading DNA polymerase (Invitrogen, Carlsbad, CA, USA) and sequenced ends of the fragments using the primers used for PCR. Resulting gaps were iteratively filled in further rounds of sequencing using new individual-specific primers flanking the gaps. The reads were aligned to the *Co.aspersum* sequence as reference and combined into a single 14070 bp contig. The sequence is available at GenBank (MK347426).

We used MITOS (Bernt et al. 2013b) to annotate the genome sequence, which identified the full set of the expected 2 rRNA, 22 tRNA, and 13 protein-coding genes (Table 1). However, we made manual adjustments to the MITOS output regarding the limits of rRNA and protein-coding genes, which were inconsistent between the MITOS annotation and the *Ce.nemoralis*, *Co.aspersum*, and *Cy.obtusus* RefSeq entries. To be more specific, we looked for start- and stop-codons whose positions would be compatible between *H.pomatia* and the other three species, and considered also the positions of adjacent genes. However, as gene overlap and alternative start- and incomplete stop-codons may occur in invertebrate mitogenomes (Bernt et al. 2013a) including land snails (Gaitán-Espitia et al. 2013), the start and end positions of the genes remain tentative until sequences of the coded proteins and rRNAs are known. The 16S gene was not recovered in one piece by MITOS, probably because the structure towards the 3’ end was too variable; so we combined the two fragments.

**Table 1.** NCBI Transcriptome Shotgun Assembly database accession numbers of the *Cepaeanemoralis* sequences used in analysis.

*cox1*	GFLU01084822
*nd6*	GFLU01007552
*nd5*	GFLU01092360
*nd1*	GFLU01092360
*nd4L*	GFLU01092360
*cyb*	GFLU01076837
*cox2*	GFLU01076837
*atp6*	GFLU01122686
*nd3*	GFLU01014131
*cox3*	GFLU01122685
*nd4*	concatenated from GFLU01122684, GFLU01122687, GFLU01122688

### Phylogenetic analyses

For the phylogenetic analysis we used a concatenated alignment of 12 protein-coding genes, excluding the short and fast evolving *atp8*. From annotated, previously published sequences (accession numbers NC_001816, NC_021747 and NC_017872), we extracted the individual genes following the annotation in the NCBI RefSeq database. However, previous studies, which used the sequence of *Ce.nemoralis* in mitogenomic phylogenetic analyses, repeatedly resulted in a spuriously long branch for this species (Knudsen et al. 2006, González et al. 2016, Minton et al. 2016, Groenenberg et al. 2017). A closer examination of the mitogenome sequence and translation of its protein coding regions revealed a very low quality of the sequence (see also White et al. 2011). Compared to newer *Ce.nemoralis* sequences there seemed to be frequent indels and suspect amino-acid changes were found in translated sequences. The very high number of errors in the sequence probably stems from the complex way it was obtained, which involved two rounds of cloning, and generally the immature methods of that time (Terrett et al. 1996). Therefore, we replaced the sequences of protein-coding genes of *Ce.nemoralis* by data retrieved from a transcriptome shotgun assembly (TSA) (Kerkvliet et al. 2017; NCBI BioProject PRJNA377398; see Table 1). To extract the mitochondrial genes from the transcriptome data, we performed for each gene a BLASTn search limited to *Ce.nemoralis* with the respective part of NC_001816 as the query in the NCBI TSA database. The sequence of *T.pisana* (MH362760) was not annotated, so we identified the individual genes by alignment with the extracted gene sequences of the other four species.

We performed the phylogenetic analyses in three stages, staring with the analysis of the nucleotide sequences. The protein coding genes were aligned with TRANSLATORX (Abascal et al. 2010), which aligns nucleotide sequences based on their amino acid translations. TRANSLATORX was run with the MAFFT 5 aligner (Katoh et al. 2005) and the invertebrate mitochondrial genetic code; other settings were left at their defaults. The resulting alignments were trimmed to a common length of the sequences. We specified a partitioning scheme consisting of the three codon positions and used IQTREE 1.6.5 to test the scheme against simpler scenarios, select substitution models (Kalyaanamoorthy et al. 2017) and to perform a maximum likelihood phylogenetic analysis (Nguyen et al. 2015, Chernomor et al. 2016). The model selection suggested the TIM2+F+I+Γ4, TVM+F+Γ4, and TIM2+F+Γ4 models for the three partitions. We used standard bootstrap and Shimodaira-Hasegawa-like approximate likelihood ratio tests (SH-aLRT; Guindon et al. 2010; both with 1000 replicates) to calculate branch supports. Then we translated the alignment into amino acid sequences, leaving it a single partition with 417 parsimony informative sites out of the total of 3489 sites, and run the model selection (selecting mtZoa+F+Γ4 as the best fit substitution model; Rota-Stabelli et al. 2009) and phylogenetic analysis as above.

The resulting topologies showed substantial differences in branch lengths between taxa. To explore if the resulting topology was influenced by long-branch attraction (LBA), we employed a site-heterogeneous mixture model using posterior mean site frequencies (PMSF; Wang et al. 2018a). This approach has been shown to effectively overcome LBA, and even to be somewhat prone to long-branch repulsion. Thus, if a grouping is due to LBA, this approach is likely to reveal that. We calculated the PMSF under the mtZoa+C60+F+Γ4 mixture model. As a guide tree we used successively all the three alternative topologies in the western group: *Cornu*+(*Theba*+*Cepaea*), (*Cornu*+*Theba*)+*Cepaea*, and (*Cornu*+*Cepaea*)+*Theba*.

Finally, to account for differences in nucleotide composition, we performed a Bayesian phylogenetic analysis of the nucleotide data with a branch-heterogeneous model allowing branches to have distinct compositions as implemented in P4 1.0 (Foster 2004; http://p4.nhm.ac.uk/). We performed the analysis in two runs of 1,000,000 generations with four heated chains each, using the same partitioning scheme as with IQTREE and the closest possible substitution models. For each data partition we allowed for three different nucleotide compositions to be assigned to tree branches randomly. We sampled each 1000^th^ generation and discarded the first 20% of samples of each run as burn-in. To account for differences in results caused by the use of different inference algorithms in the methods, we repeated the analysis in P4 also assuming composition homogeneity.

## Results

We have sequenced a complete mitogenome of *H.pomatia*, from a specimen representative of a common central-European lineage (Korábek et al. 2018) of this broadly distributed snail species. The length and gene content and order (Table 2) correspond with those of mitogenomes of *Ce.nemoralis*, *T.pisana* and *Co.aspersum*, the other Helicinae species with available mitogenome sequences.

**Table 2.** Annotation of the mitogenome of *Helixpomatia*. The plus/minus strand refers to the position of the *cox1* gene.

Gene	Start position	Start codon	End position	Stop codon	Strand
*cox1*	1	ATG	1530	TAA	plus
*trnV*	1527		1589		plus
*rrnL (16S)*	1657		2555		plus
*trnL1*	2566		2626		plus
*trnA*	2630		2691		plus
*nd6*	2692	TTG	3180	TAA	plus
*trnP*	3177		3239		plus
*nd5*	3285	ATG	4955	TAA	plus
*nd1*	4970	ATA	5842	TAG	plus
*nd4L*	5842	GTG	6118	T--	plus
*cyb*	6119	ATG	7190	T--	plus
*trnD*	7234		7285		plus
*trnC*	7286		7345		plus
*trnF*	7346		7404		plus
*cox2*	7405	ATG	8071	T--	plus
*trnY*	8073		8119		plus
*trnW*	8121		8180		plus
*trnG*	8186		8233		plus
*trnH*	8237		8296		plus
*trnQ*	8296		8355		minus
*trnL2*	8356		8411		minus
*atp8*	8405	ATA	8578	TAG	minus
*trnN*	8579		8638		minus
*atp6*	8637	ATG	9290	T--	minus
*trnR*	9291		9349		minus
*trnE*	9349		9411		minus
*rrnS (12S)*	9479		10173		minus
*trnM*	10173		10235		minus
*nd3*	10241	TTG	10581	T--	minus
*trnS2*	10579		10629		minus
*trnT*	10632		10693		minus
*cox3*	10693	ATG	11472	TAA	minus
*trnS1*	11664		11719		plus
*nd4*	11720	ATA	13027	TAA	plus
*trnI*	13030		13090		plus
*nd2*	13091	GTG	14021	T--	plus
*trnK*	14022		14070		plus

**Figure 1.**
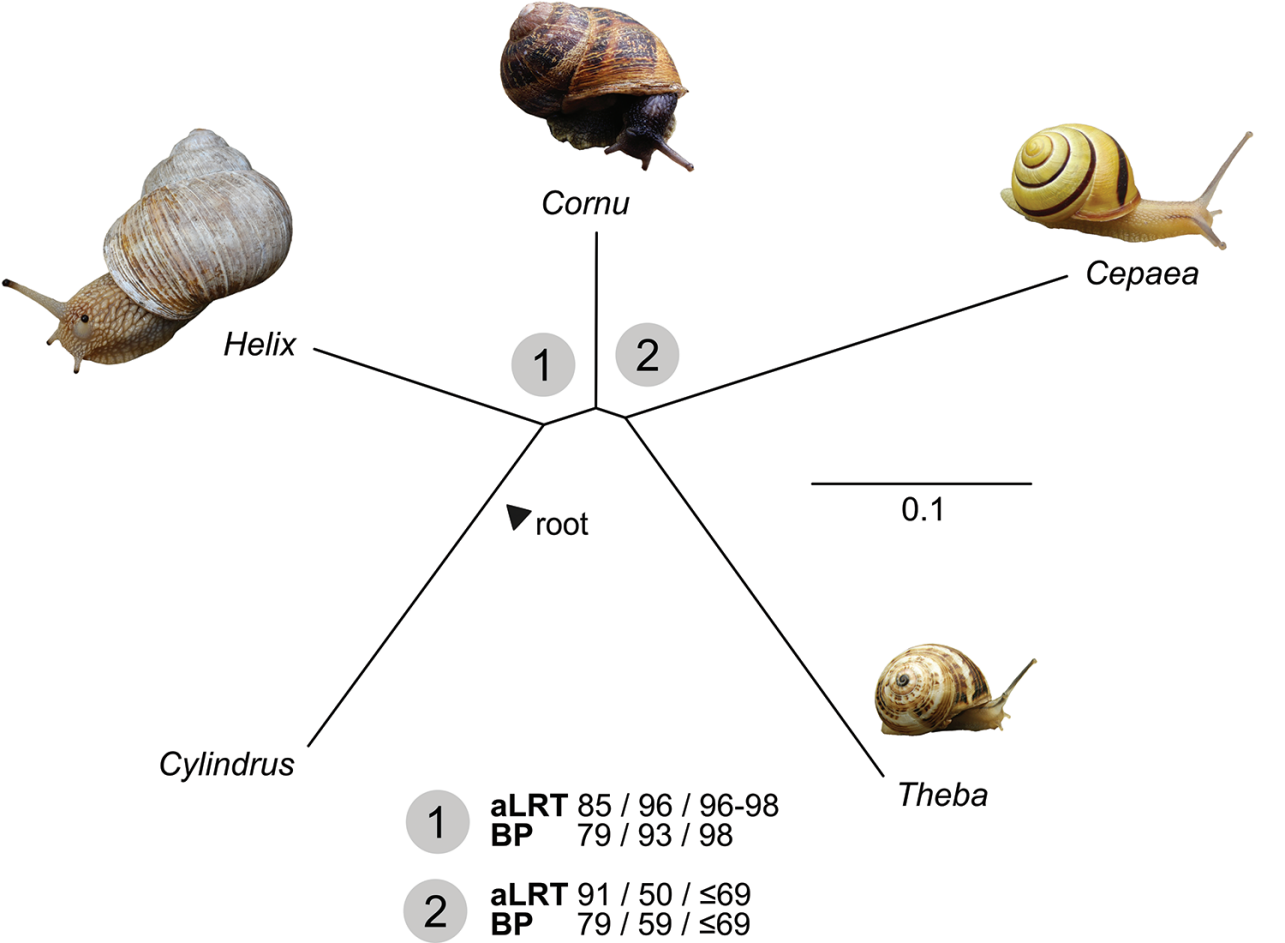
The inferred phylogenetic relationships between the five helicid mitogenomes available. Branch supports are given for maximum likelihood analyses based on nucleotide data, amino acid data under homogeneous model, and amino acid data under site-heterogeneous model (in this order). The latter was run under alternative settings, see details in the text. Shimodaira–Hasegawa-like approximate likelihood ratio test (SH-aLRT) and standard bootstrap percentages (BP) are reported. Values of SH-aLRT >90 % and BP >75 % are considered positive support. *Cylindrus* is included as an outgroup.

The phylogenetic analyses in all cases recovered the expected split between the eastern *Helix* and the western group of *Theba*, *Cepaea*, *Cornu*, assuming *Cylindrus* as an outgroup (Figure 1). This grouping received equivocal support in the analysis based on the nucleotide data, but it increased with amino acid data and further when using site-heterogeneous model. The support thus increased as the analyses got less sensitive to substitution saturation and among site heterogeneity. In the western group, the results united *Theba* and *Cepaea* to the exclusion of *Cornu*, but this branch received positive support only from bootstrap with the nucleotide data. Especially analyses of the amino-acid alignment with the site-heterogeneous model based on alternative guide trees resulted in very low support for the branch uniting *Cepaea* and *Theba*. The Bayesian analysis of nucleotide data with P4 recovered the same topology as the maximum likelihood analysis but with full support, regardless whether homo- or heterogeneous model was used.

## Discussion

Because *Theba* and *Cepaea* had the longest branches in the tree, which had short internal branches, and there is no independent indication of the relationship between *Theba* and *Cepaea*, we suspected the result to be caused by the LBA (Bergsten 2005). *Theba* is annual to biannual and semelparous (Heller 1982, Cowie 1984), unlike the other helicids, which are more long-lived (Pollard 1975, Bisenberger et al. 1999; Ansart et al. 2009). The genus may generally cause problems in phylogenetic inference if the difference in life history results in differece in substitution rate (Thomas et al. 2010; cf. Saclier et al. 2018). In addition, analysis of the nucleotide composition of the five taxa revealed substantial differences, where *Cornu* (GC 30.5%) and *Cepaea* (GC 41.6%) differed the most, which could obscure potential relationship between the two species. The low support with presumably more robust methods suggests that the relationship between *Cepaea* and *Theba* could be an artefact.

Our results are consistent with the hypothesis that Helicinae are principally divided into a western (mainly Iberia, western Maghreb, Macaronesia) and an eastern (Caucasus, Anatolia, Greece) group (Neiber and Hausdorf 2015). The split between these two lineages probably occurred no later than during the Late Eocene‒Early Oligocene (Neiber and Hausdorf 2015), but the east-west pattern in the distribution of the two lineages persists despite 30 million years having elapsed since the split. Only two *Helix* species naturally represent the eastern group west of ca 9°E (one in Europe, one in Africa; Neubert 2014, Korábek et al. 2018), and two species of *Cepaea* (Europe; Welter-Schultes 2012) and few species of *Eremina* Pfeiffer, 1855 (northern Africa; Ali et al. 2016) represent the western group east of 18°E.

Despite analysing a substantially higher number of genes than Razkin et al. (2015) and Neiber and Hausdorf (2015), we also could not resolve the relationships between *Cornu*, *Cepaea* and *Theba*. We assume that additional lineages should be analysed in order to resolve all the major phylogenetic and biogeographic problems within the western branch of Helicinae. These include *Macularia* Albers, 1850, whose phylogenetic position is equally problematic as that of *Theba* (Nordsieck 1987; Schileyko 2006; Neiber and Hausdorf 2015), the closest relative of *Theba* (perhaps *Eremina*, but see Holyoak et al. 2018), and the type genera of the tribes Otalini and Allognathini. Nevertheless, it is likely that only analyses of multiple nuclear loci will yield robust estimates of the basal relationships of Helicinae due to high and lineage-specific evolutionary rates of the mtDNA and the saturation of nucleotide substitutions.
